# Treatment and management of *Salmonella* prostatitis in a heartworm‐positive intact male dog: a case report

**DOI:** 10.1186/s12917-021-02836-7

**Published:** 2021-03-30

**Authors:** John N. Hertzer, Madeline Fujishiro, Sara D. Lawhon, Kate E. Creevy

**Affiliations:** 1 Department of Small Animal Clinical Sciences, Texas A&M University, 77843-4474 College Station, TX USA; 2 Las Vegas Veterinary Specialty Center, 89147 Las Vegas, NV USA; 3 Department of Veterinary Pathobiology, Texas A&M University, 77843-4467 College Station, TX USA

**Keywords:** Salmonella prostatitis, salmonella zoonosis, Zoonotic prevention

## Abstract

**Background:**

*Salmonella* spp. represent a significant zoonotic concern to pregnant owners as infection can cause septic abortions and post-partum illness. Enteric salmonellosis is well documented in canines however urinary salmonellosis is rarely described and *Salmonella* prostatitis has never been described in dogs.

**Case presentation:**

This case report describes the diagnosis and management of a five-year-old, intact male Labrador Retriever mix dog that was diagnosed with *Salmonella* prostatitis among other comorbidities including heartworm infestation. Additionally, mitigation of zoonotic spread is emphasized as one of the owners was six months pregnant at the time of diagnosis.

**Discussion:**

The pathogenesis of *Salmonella* prostatitis is unknown but explanations pertaining to enteric salmonellosis, such as the lifestyle and stress of living as a stray may have contributed and contamination from an enteric infection may have also been possible. Several recommendations were made to reduce the likelihood of zoonotic transmission including frequent hand washing, avoidance of the patient’s mouth, change in location of where the patient was fed, the use of an isolated area outside for urination and defecation, and the use of dilute bleach to clean areas soiled by the patient’s bodily fluids. Monitoring of the prostatic infection was facilitated with prostatic wash instead of urine culture. This decision was made as prostatic infections have been shown to intermittently shed bacteria into the urine, leading to possible false negative urine cultures and potential catastrophic zoonotic infection.

## Background

*Salmonella enterica* is estimated to cause approximately 1.2 million human illnesses, 22,000 hospitalizations and 425 deaths annually in the United States, at an annual cost of $3.3 billion [1–3]. In pregnant women, salmonellosis has been shown to cause septic abortion [4], post-abortion and post-partum septicemia [5], and neonatal septicemia [4]. Dogs are well-documented enteric hosts for *Salmonella enterica* with a prevalence of 2.5 %, and are thus a potential source of zoonotic transmission [6–8]. Dogs are usually subclinical carriers and are less likely to develop clinically significant disease; however, they can shed the bacteria through feces intermittently for six weeks or more post infection [9]. Possible clinical signs associated with salmonellosis in dogs include anorexia, fever, or diarrhea with isolated reports of meningoencephalitis, diskospondylitis, and acute hepatic necrosis [7, 10–12]. Urinary salmonellosis, however, has rarely been described in dogs [13] and to the authors’ knowledge, confirmed *Salmonella* spp. prostatitis has not been described in the dog. This case report describes the challenges associated with the management of a novel site of salmonellosis in a heartworm-positive intact male dog living with a pregnant owner.

## Case presentation

A stray intact male Labrador Retriever mix, estimated to be five years old, was adopted and was promptly evaluated by a veterinarian for lethargy and poor appetite. Physical examination noted ectoparasitism and asymmetric left-sided facial muscle atrophy. Complete blood count (CBC) revealed severe thrombocytopenia (0 platelets/microliter [µL], confirmed via blood smear) and chemistry profile revealed mild hyperglobulinemia (5.4 gram [g]/deciliter [dL]). Urinalysis obtained via cystocentesis revealed pyuria with 6–20 white blood cells (WBC)/high power field (hpf). An in-house infectious disease ELISA^1^ was reported as heartworm antigen positive, *Ehrlichia* spp. antibody positive, and *Anaplasma* spp. antibody positive. Thoracic radiographs revealed a mild bronchointerstitial pulmonary pattern and mild pulmonary artery enlargement.

The dog was prescribed doxycycline at 5.7 milligrams (mg)/kilogram (kg) *per os* (PO) twice a day (BID) for 28 days, sulfamethoxazole and trimethoprim (SMZ-TMP) at 27.3 mg/kg PO once daily for 14 days, and prednisone at 1.1 mg/kg PO BID. He was also administered an anthelmintic and prescribed monthly heartworm, flea, and tick prevention.

At a two-week re-evaluation, his appetite had improved, and the owner reported he was polyuric and polydipsic. CBC revealed an improvement in his thrombocytopenia (156,000 platelets/µL) and his prednisone dose was tapered by 25 %. At his recheck three weeks later, his urine was reported to be foul-smelling. His CBC revealed a worsening thrombocytopenia (117,000 platelets/µL) and chemistry profile was unremarkable. Urinalysis reported pyuria and rod-shaped bacteria. Urine obtained via cystocentesis was submitted for aerobic culture and amoxicillin/clavulanic acid^2^ was prescribed at 13.8 mg/kg PO BID while results were pending. Due to his progressive thrombocytopenia, his prednisone was returned to the 1.1 mg/kg PO BID dose and he was prescribed azathioprine at 2.4 mg/kg PO once daily. Urine culture results reported > 100,000 organisms/milliliter (mL) of *Salmonella* spp. and he was prescribed amoxicillin 25.3 mg/kg PO BID for 28 days (amoxicillin/clavulanic acid was discontinued). Ten days later, he was evaluated for a three-day history of progressive unilateral (left-sided) epistaxis and observed “red urine”. CBC revealed a severe thrombocytopenia (23,000 platelets/µL); clotting times (prothrombin time and partial thromboplastin time) were within normal limits. He was referred to the Texas A&M University Veterinary Medical Teaching Hospital (TAMU-VMTH) for further evaluation.

At TAMU-VMTH six weeks after original adoption, problems and considerations for this patient have been summarized in Table 1 and included:

**Table 1:** Summary of clinical problems with associated action steps

Clinical Finding	Source	Action
**Thrombocytopenia**		
0 plt/µL	RDVM	Begin prednisone 2.2 mg/kg/day PO
156,000 plt/µL	RDVM	Decrease prednisone by 25 %
117,000 plt/µL	RDVM	Increase prednisone to 2.2 mg/kg/day PO; begin azathioprine 2.4 mg/kg PO daily
23,000 plt/µL	RDVM	Refer to TAMU-VMTH
171,000 plt/µL	TAMU-VMTH	Discontinue azathioprine; decrease prednisone by 50 %
102,000 plt/µL	TAMU-VMTH	Begin cyclosporine at 4.7 mg/kg/day PO; begin Yunnan Baiyao 0.5 g PO BID; increase prednisone by 25 %
160,000 plt/µL	TAMU-VMTH	Taper cyclosporine to discontinuation; discontinue Yunnan Baiyao
**Vector-borne diseases**		
* Ehrlichia* spp. antibody seropositivity	RDVM	Begin doxycycline 5.7 mg/kg PO BID
* Anaplasma* spp. antibody seropositivity	RDVM	Begin doxycycline 5.7 mg/kg PO BID
* D. immitis* antigen seropositivity	RDVM	Begin doxycycline 5.7 mg/kg PO BID
Echocardiographic confirmation of heartworm infestation	TAMU-VMTH	Administer melarsomine three-dose protocol (2.5 mg/kg/dose)
**Urinary tract infection**		
6–20 WBC/hpf on UA	RDVM	Begin empiric sulfamethoxazole- trimethoprim 27.3 mg/kg PO daily x 14d
bacteriuria / pyuria on UA	RDVM	Begin empiric amoxicillin/clavulanic acid 13.8 mg/kg PO BID
> 100,000 organisms/mL of *Salmonella* spp. from urine culture	RDVM	Discontinue amoxicillin/clavulanic acid; begin amoxicillin 25.3 mg/kg PO BID x 28d
4 colonies of *Salmonella* spp. from prostatic wash culture and susceptibility	TAMU-VMTH	Begin enrofloxacin 10.6 mg/kg PO daily x 28d; begin doxycycline 10.1 mg/kg PO BID x 28d; initiate zoonosis precautions in the home
Negative urine culture after 14 days of enrofloxacin / doxycycline	TAMU-VMTH	No action
> 100,000 organisms/mL of *Salmonella* spp. from prostatic wash culture	TAMU-VMTH	Begin marbofloxacin 3 mg/kg PO daily through 2 weeks post-castration
Negative prostatic wash culture 10 d after stopping marbofloxacin	TAMU-VMTH	

Clinical data and action steps are in chronological order within each problem category

*RDVM* referring veterinarian. *TAMU-VMTH* Texas A&M University Veterinary Medical Teaching Hospital

- heartworm infestation (doxycycline had been begun prior to adulticide therapy).
- *Ehrlichia* spp. and *Anaplasma* spp. seropositivity (doxycycline had been begun).
- intermittent epistaxis and gross hematuria (possibly resulting from systemic infectious or inflammatory vasculitis or thrombocytopenia, or from two local diseases).
- varying thrombocytopenia (while only one measured value was severely low, the presence of clinical bleeding increased suspicion of true severity; immunosuppression with prednisone and azathioprine had been begun).
- facial asymmetry (relationship with concurrent epistaxis was unclear).

*Salmonella* culture-positive urine (concurrent hematuria; in an intact male dog, complicating prostatitis was presumed; poor anesthetic candidate for castration until heartworm adulticidal therapy was complete).

At the time of the TAMU-VMTH visit, it was also revealed that one of the owners was approximately six months pregnant with the couple’s first child, raising additional concerns regarding treatment and precautions of the diagnosed *Salmonella* infection. Once the diagnosis of salmonellosis was determined, the owners were instructed to have the dog urinate/defecate in an isolated portion of the yard that the other housemate, a 10-year-old castrated male German Shepherd, could not access. The male owner was instructed to remove the feces while wearing gloves. If any urination or defecation occurred within the house, the male owner was instructed to use gloves while removing organic material and to then clean the area with a bleach-based solution. It was recommended to move the patient’s water and food bowls to a separate, low-traffic area of the house to minimize *Salmonella* exposure in the kitchen. Both owners were also instructed to wash their hands frequently.

Static asymmetric left-sided facial muscle atrophy and pain on prostatic palpation were found. CBC revealed 171,000 platelets/µL and chemistry profile reported severe elevations in liver enzymes suspected to be secondary to azathioprine hepatotoxicity. Blood pressure was normal and thoracic radiographs confirmed previous findings consistent with heartworm infestation. Abdominal ultrasound revealed a few small ill-defined hypoechoic liver nodules (suspect vacuolar hepatopathy), slightly small adrenal glands bilaterally (likely secondary to exogenous corticosteroids), and a mildly enlarged irregular heterogenous prostate (suspect prostatitis). An echocardiogram was performed and identified one heartworm in the distal right pulmonary artery with no significant cardiac remodeling or evidence of pulmonary hypertension. A prostatic wash cytology showed mild atypical epithelial cells (suspect reactive change) and culture of prostatic wash fluid isolated four colonies of *Salmonella* species. The isolate was initially identified as *Salmonella* species using matrix-assisted laser desorption–time of flight (MALDI-TOF) mass spectrometry using the Biotyper™^3^with flexControl v3.4 build 135.14 software. The isolate from this culture was submitted to the National Veterinary Service Laboratories (NVSL) in Ames, Iowa for serotyping and was determined to be *Salmonella enterica* serovar III 41:z4,z23:-. Subsequently the isolate was sequenced using Illumina MiSeq and confirmed to be *Salmonella enterica* subsp. *arizonae* serovar 41:z4,z23:-. The isolate was then sequenced using 2 × 300 bp paired-end reads^4^ and confirmed to be *Salmonella enterica* subsp. *arizonae* serovar 41:z4,z23:- using SeqSero, in agreement with the NVSL report [14]. Details of the sequence analysis have been previously published [15]. Based on culture and susceptibility results, enrofloxacin at 10.6 mg/kg PO once daily for 28 days was prescribed, as well as another course of doxycycline at 10.1 mg/kg PO BID for 28 days and reduction of the prednisone dose 1.0 mg/kg PO once daily. Azathioprine and amoxicillin were discontinued.

Magnetic resonance imaging (MRI) and computed tomography (CT) of the brain/skull performed one week later did not reveal a cause for the dog’s facial asymmetry and left-sided epistaxis. Cerebrospinal fluid (CSF) analysis revealed no cytological abnormalities. A von Willebrand factor antigen assay (vWF:Ag) returned a result of 33 %, with < 50 % being consistent with an abnormal result or carrier range. Yunnan Baiyao at 0.5 g PO BID was prescribed. Because CBC revealed progressive thrombocytopenia (102,000 platelets/µL), cyclosporine at 4.7 mg/kg PO once daily was prescribed and prednisone was increased to 1.25 mg/kg PO BID. No further episodes of epistaxis were reported, and Yunnan Baiyao was eventually discontinued without any adverse bleeding events.

Two weeks after initiating enrofloxacin, urine collected by cystocentesis yielded no bacterial growth on quantified urine culture. CBC revealed 160,000 platelets/µL and chemistry profile showed moderate improvement of the hepatopathy. Immune-mediated platelet destruction was considered less likely at this point due to resolution of bleeding events, and persistence of only moderate, fluctuating thrombocytopenia. Cyclosporine was slowly tapered without a relapse of severe thrombocytopenia and prednisone was tapered once heartworm adulticide therapy was completed. The platelet count remained mildly low throughout treatment for heartworm disease and *Salmonella* prostatitis. An underlying etiology was never confirmed.

Approximately one month later when the liver enzyme elevation had resolved, the dog returned for the first heartworm adulticide treatment with melarsomine at 2.5 mg/kg as recommended by the American Heartworm Society [16]. A prostatic wash culture was also performed (two weeks after completion of enrofloxacin). The culture grew > 100,000 organisms/mL of *Salmonella* spp. It was unknown if the prostate had remained persistently infected (as only a urine culture without prostate wash had been performed at mid-treatment recheck) or if repeated re-infections of the prostate were occurring. The isolate was submitted to NVSL and WGS was performed and the serotype was the same as that previously isolated from the patient’s urine (serotype 41:z4,z23:-). Fecal *Salmonella* PCR to detect the *spaQ* was performed on three fecal samples collected 24 h apart and all three samples were positive. An isolate recovered from the feces was submitted to NVSL and determined to be *Salmonella enterica* subsp *arizonae* serovar 41:z4,z23:-, the same serovar isolated from the prostate. The PCR was performed as previously described [17] with the modifications that the PCR was performed using a different real time PCR thermocycler^5^ and PCR reagents.^6^A positive control of genomic DNA from a clinical strain of *Salmonella enterica* subspecies *enterica* serovar Typhimurium was utilized. Negative control PCR reactions included reactions with either genomic DNA from *Escherichia coli* strain ATCC 29,522 or with no DNA added to the reaction. Identification was confirmed with serotyping by the National Veterinary Services Laboratory.

Due to the challenge of curing bacterial prostatitis in an intact male dog, castration was planned as soon as anesthesia could safely be performed after heartworm adulticide therapy. Based on culture and sensitivity results, and considerations for adequate prostatic and fecal penetration, marbofloxacin was prescribed at 3 mg/kg PO once daily. In an attempt to decrease environmental contamination of the home environment with *Salmonella*, antimicrobial therapy was planned for the duration of his heartworm therapy. Heartworm therapy with two additional doses of melarsomine was completed six weeks later, after delivery of the owners’ new baby. A prostatic wash culture was performed, and *Salmonella* was isolated after broth enrichment (serotype 41:z4,z23:-). Fecal *Salmonella* PCR was also performed and was negative on all three samples. Marbofloxacin was continued.

One month later when the dog was considered a more stable anesthetic candidate, castration was performed. Desmopressin 0.1 mg was administered subcutaneously 30 min prior to surgery due to the low vWF:Ag and surgery was completed without incident. A prostatic wash culture wash also performed and reported no growth. Marbofloxacin was continued for two additional weeks. Ten days after stopping marbofloxacin, a final prostatic wash culture was performed and reported no growth.

## Discussion and conclusions

In this case, the source of the *Salmonella* infection is unknown. Several lifestyle factors of this patient suggest possible sources. First, the presumed predatory and scavenger lifestyle while living as a stray may have increased the exposure risk to *Salmonella*. *Salmonella enterica* subspecies *arizonae* is a commensal organism in reptiles, such as snakes, and has been associated with disease in poultry [18, 19]. It is possible that this patient had prior exposure to or ingestion of reptiles while living as a stray. It has been reported that dogs in Texas animal shelters had an overall prevalence of fecal *Salmonella* of 4.9 %, and the rate tended to be higher among dogs who arrived at the shelter as strays than among those who had been surrendered; importantly, *Salmonella*-positive feces in this study were most often normal in appearance [20]. Additionally, the physiologic stress of living as a stray may have made this patient more susceptible to infection. Chronic stress increases exposure to catecholamines and glucocorticoids, which can influence humoral immunity as well as intestinal flora growth, biofilm formation, and motility, potentially allowing for pathogenic bacterial colonization within the intestines [20, 21]. These explanations are commonly applied to enteric salmonellosis. The pathogenesis of this patient’s *Salmonella* prostatitis is unknown, but it is plausible that the same risks of exposure and impaired immune clearance are relevant to salmonellosis outside the gastrointestinal tract as well. It is also plausible that this patient’s urinary tract infection was a result of contamination from an original enteric infection.

Castration is generally recommended as adjunct treatment for prostatitis in dogs [22–24]. In the prospective study by Cowan et al. [24], castration reduced the period of positive prostatic culture by more than four weeks. Additionally, castration was also shown in that study to significantly reduce the amount of bacteria isolated from the urine when compared to intact males [24]. With these facts in mind, it was imperative in this case to castrate the patient as soon as possible to help prevent zoonotic spread of the isolated *Salmonella.* Until very recently, canine castration has only been described while under general anesthesia mainly due to the large amount of perceived nociception associated with the procedure [25]. In non-shelter settings, however, heartworm-infested patients are considered to be at increased risk of anesthetic complications [26]. This increased risk is partly due to the reduction in pulmonary vascular resistance, which can result in post-operative pulmonary thromboembolism [26]. Because of this increased risk for this patient, castration was scheduled after completion of heartworm adulticidal therapy.

Regardless of when castration would occur, it was imperative to implement a protocol at home to help prevent zoonotic spread to the pregnant owner. The first recommendation was to have the non-pregnant owner take over feeding of the patient. Additionally, frequent hand washing was recommended for both owners, especially after interacting with the patient in any way and prior to eating a meal [27]. If the pregnant owner were to touch any of the patient’s bodily fluids, it was recommended to wear gloves followed by careful hand washing. Avoidance of the patient’s mouth was also recommended to prevent contact with saliva [27]. The owners were also asked to feed the patient in a different room than the kitchen to reduce the amount of time the patient spent in areas where preparation and consumption of human food occurred. In a recent *Salmonella* outbreak in children that was associated with pet food, one of the identified risk factors pertained to where pets were fed [28]. When outside, it was recommended to have the patient urinate and defecate in a separate, isolated portion of the yard not used by other pets, and where the non-pregnant owner could promptly remove any feces; as mentioned previously, even non-diarrheic canine feces can be positive for *Salmonella* [20, 29]. Lastly, when the non-pregnant owner needed to clean an area soiled by the patient’s bodily fluids, disinfection with household bleach, diluted 1:10 to 1:20 with water, was recommended after any organic material had been removed [29].

In this case, it was important to monitor infection status until clearance of *Salmonella* could be confirmed. Options for monitoring included urine culture and fecal PCR, as well as prostatic wash cultures. Prostatic wash was chosen for several reasons. It has been shown that prostatic infections can intermittently shed bacteria into the urine, leading to false negatives if urine culture alone were used [24]. In this case, a false negative urine culture at the ten-day post-antibiotic recheck would have increased the risk of potentially catastrophic zoonosis. In regards to fecal PCR, currently it is recommended that if a PCR-positive result for *Salmonella* is obtained, a fecal culture should be performed to identify the organism [29]. In this case, organism identification by fecal culture following a positive fecal PCR was considered to be less clinically relevant than identification of the organism in the urinary tract. Similarly, it was not clear that a negative fecal PCR would indicate a concurrent clearance of the urinary infection. Confirmed *Salmonella* spp. prostatitis has not previously been described in the dog. In the dog of this case, treatment of heartworm disease, followed by castration, facilitated successful resolution of *Salmonella* prostatitis in a dog living with a pregnant owner. This report highlights the importance of using prostatic wash to monitor prostatic infection as intermittent bacteriuria was observed. Additionally, this report provides a practical review on the importance and logistics of reducing the risk of zoonotic infection for dog owners.

## Data Availability

All data generated or analyzed during this study are included in this published article.

## Abbreviations

Spp.: Species, plural
CBC: Complete blood count
WBC: White blood cells
ELISA: Enzyme-linked immunoassay
PO: *per os* (by mouth)
BID: *bis in die* (twice a day)
TAMU-VMTH: Texas A&M University Veterinary Medical Teaching Hospital
MALDI-TOF: Matrix-assisted laser desorption–time of flight.
NVSL: National Veterinary Science Laboratory
MRI: Magnetic resonance imaging
CT: Computed tomography
vWF:Ag: :Von Willebrand Factor antigen assay
WGS: Whole genome sequencing
PCR: Polymerase chain reaction

## Footnotes

1 4Dx SNAP test; Idexx, Inc., Westport ME.

2 Clavamox, Zoetis Petcare, Parsippany-Troy Hills, NJ.

3 Bruker Daltonics, Billerica, MA]

4 MiSeq™, Illumina, San Diego, CA.

5 ABI 7900HT Fast real time PCR machine (Applied Biosystems)]

6 Taqman Fast Universal PCR Master Mix 2X, No Amperase UNG, ThermoFisher Scientific Waltham, MA.
